# High-Efficiency Adsorption Removal of Congo Red Dye from Water Using Magnetic NiFe_2_O_4_ Nanofibers: An Efficient Adsorbent

**DOI:** 10.3390/ma18040754

**Published:** 2025-02-08

**Authors:** Hellen C. T. Firmino, Emanuel P. Nascimento, Keila C. Costa, Luis C. C. Arzuza, Rondinele N. Araujo, Bianca V. Sousa, Gelmires A. Neves, Marco A. Morales, Romualdo R. Menezes

**Affiliations:** 1 Laboratory of Materials Technology (LTM), Department of Materials Engineering, Federal University of Campina Grande, Campina Grande 58429-900, Brazil; emanueluepb@gmail.com (E.P.N.); keilaconceicaocosta@gmail.com (K.C.C.); luisarzuza179@gmail.com (L.C.C.A.); rondinelenunes93@gmail.com (R.N.A.); gelmires.neves@ufcg.edu.br (G.A.N.); 2 Graduate Program in Materials Science and Engineering, Federal University of Campina Grande, Campina Grande 58429-900, Brazil; 3 Department of Chemical Engineering, Federal University of Campina Grande, Campina Grande 58429-900, Brazil; biancavianaeq@gmail.com; 4 Department of Theoretical and Experimental Physics, Federal University of Rio Grande do Norte, Natal 59078-970, Brazil; marco.moralestorres@gmail.com

**Keywords:** water treatment, pollution, SBS method, wastewater, magnetic NiFe_2_O_4_ nanofibers

## Abstract

The pollution caused by organic dyes in water bodies has become a major environmental issue, and removing such pernicious dyes presents an immense challenge for the scientific community and governments. In this study, a sorbent based on nickel ferrite (NiFe_2_O_4_) fibers was fabricated by the solution blow spinning (SBS) method for the adsorptive removal of anionic Cong red (CR) dye. The cubic–spinel structure and the magnetic and porous nature of NiFe_2_O_4_ were confirmed by XRD, magnetometry, BET, and SEM analyses. The saturation magnetization confirmed the magnetic nature of the fibers, which favorably respond to an external magnetic field, facilitating separation from a treated solution. The sorption kinetics of CR on NiFe_2_O_4_ were best described by the pseudo-second-order model, while sorption equilibrium agreed best with the Freundlich, Langmuir, Sips, and Temkin isotherm models, suggesting a complex mechanism involving chemisorption, monolayer coverage, and heterogeneous adsorption. The NiFe_2_O_4_ fibers annealed at 500 °C showed a high CR removal efficiency of ~97% after only 30 min. The sorbent’s porous structure and high specific surface area were responsible for the improved removal efficiency. Finally, the results indicated the potential of the NiFe_2_O_4_ fibers in the remediation of water contaminated with Congo red dye.

## 1. Introduction

The printing, plastic, leather, cosmetic, and textile industries frequently use Congo red (CR), a diazo, anionic, water-soluble dye with high chemical stability, which is highly toxic to humans and aquatic life [1]. These industries, together, are expected to account for a market value of over USD 2 trillion in 2025 [2], which represents an enormous impact on the global economy. Additionally, in recent years, dye wastewater production has risen significantly, with over 100,000 metric tons of dyes produced annually. Approximately 10% of these dyes are released into the environment, threatening aquatic life and endangering human health. Due to insufficient industrial regulation, 10% to 15% of dyes are released into the water-based ecosystem [3]. In humans, it can cause severe damage to the kidneys, liver, central nervous system, and reproductive system, in addition to being carcinogenic, mutagenic, and allergenic [4,5]. Even in minute quantities, Congo red can be toxic [6]. Incorrect disposal of this dye, which is associated with water scarcity, leads to serious environmental and public health problems.

The need for clean water globally has forced governments and the scientific community to search for efficient water purification methods. Advanced technologies have been developed to remove water contaminants, including membrane filtration, coagulation and flocculation, ion exchange [7], bio-sourced and synthetic materials [8,9], and adsorption technologies. Among these methods, adsorption has the advantage of being a low-cost, efficient, and easy-to-implement process. The effective adsorption removal of contaminants from water can be achieved using a variety of adsorbents. The most prominent sorbents are carbon-based materials [10], mineral sorbents [11], and magnetic materials, such as nickel ferrite (NiFe_2_O_4_), magnetite (Fe_3_O_4_) [10], hematite (α-Fe_2_O_3_) [12], zinc and manganese ferrite [13]. Successful dye removal can also be achieved through biosorption with magnetotactic bacteria (MTB) [7,14]. Carbon-based materials are highly efficient adsorbents but have high production costs and limited adsorption capacity, requiring frequent regeneration, which is also challenging and costly [15]. Biosorbents are a recent class of environmentally friendly adsorbents that present magnetic properties and show potential in wastewater remediation, particularly for removing contaminants like heavy metals and organic pollutants. However, they have several drawbacks, including the limited number of contaminants they can remove or degrade effectively, the specific environmental conditions (pH, temperature, oxygen levels) they operate in, the contamination risks, and more importantly, the high costs and challenges in scaling up their production [16].

On the other hand, magnetic materials (zinc and manganese ferrite, nickel ferrite, magnetite, hematite, etc.) are effective, low-cost, and safe adsorbents presenting high stability, environmental compatibility, and magnetic properties that facilitate their separation from water [7,17]. Currently, many studies have reported on the properties and application of magnetic materials in water remediation. Recently, Debnath and Das [18] sintered Ni-ferrite nanoparticles for crystal violet dye adsorption. After 60 min of testing, 95% removal of the dye and an adsorption capacity of 19 mg/g were achieved. Patil and Behera [19] produced NiFe_2_O_4_ for the removal of thiazole yellow (TYG) and alizarin yellow (AYR) from water. The ferrite adsorbed had an efficiency of 96% and 93% in the removal of TYG and AYR, respectively, after 90 min. Starko et al. [20] evaluated Ni-Co-La for Congo red removal from water and observed an uptake capacity ranging from 43% to 98%, after 24 h, according to the adsorbate amount, 150 and 98 mg/L, respectively. Wang et al. [21] used NiFe_2_O_4_ to assist graphene in the adsorption of acridine orange. The ferrite alone adsorbed around 20 mg/g of the dye after 12 h and it was effective in magnetic separation for the reusability of the system. More recently, ZnMnFe_2_O_4_ ferrite nanoparticles synthesized by Filipović et al. [13] showed efficient removal of the anionic anthraquinone RB19 dye, with a maximum adsorption capacity of 1498.99 mg/g at pH 2 and a sorbent dose of 0.25 mg/L. This highlights the potential of NiFe_2_O_4_ in decontaminating water-containing dyes.

Nevertheless, most of these studies used nanoparticles for the adsorption of dyes, and nanoparticles are very susceptible to agglomeration, which is very detrimental to the adsorption process. The natural tendency of nanoparticles to agglomerate means that they cannot be effectively applied commercially due to their lower adsorption efficiency under the conditions of use. In terms of shape, nanofibers are less mobile in liquids than nanoparticles and less susceptible to agglomeration, maintaining high porosity and a high surface area [22]; as a result, nanofibers have greater adsorption capacity than nanoparticles. Several studies in the last year have demonstrated the higher efficiency of nanofibers in the adsorption of dyes [23,24,25,26,27]. However, few studies address the use of ferrite nanofibers as a recoverable dye adsorption system. Appiah-Ntiamoah et al. synthesized ZnO-ZnFe_2_O_4_ fibers by electrospinning for their application as a Congo red adsorbent [28]. After 160 min, 88% dye removal was achieved with an adsorption capacity of 263 mg/L. This demonstrates the potential of ferrite fibers for adsorbing dyes. However, no work has evaluated the potential of NiFe_2_O_4_ nanofibers for the adsorption removal of Congo red from water. Therefore, the objective of this study is to evaluate the adsorption process of Ni ferrite nanofibers in the removal of Congo red dye from aqueous solutions. Nanofibers were produced using the solution blow spinning (SBS) method, and their morphology, structure, and magnetic properties were also studied.

## 2. Materials and Methods

### 2.1. Synthesis of NiFe_2_O_4_ Fibers

**Figure 1:**
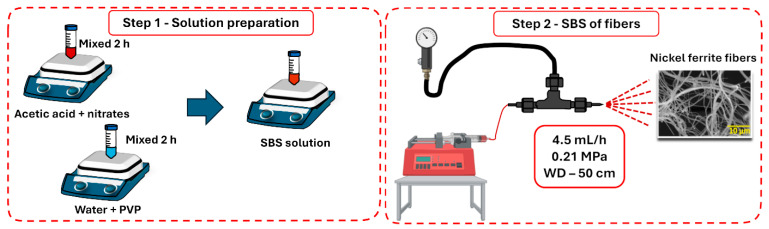
Schematic illustration of the steps in the solution blow spinning process of the nickel ferrites.

Figure 1 presents a schematic representation of the solution blow spinning method used to synthesize the magnetic NiFe_2_O_4_ fibers. A molar ratio of 2:1 (Fe:Ni) between the precursors was used in the synthesis. An amount of 1.6 g of Fe(NO_3_)_3_ (iron III nitrate nonahydrate, Sigma-Aldrich, St. Louis, MO, USA) and 0.58 g of Ni(NO_3_)_2_ (nickel II nitrate hexahydrate, Sigma-Aldrich, St. Louis, MO, USA) were added to 3 mL acetic acid (Sigma-Aldrich, St. Louis, MO, USA) and constantly stirred for 2 h at room temperature. The 12 *w/v*% PVP solution (polyvinylpyrrolidone, Mw = 1,300,000 g/mol, Sigma Aldrich, St. Louis, MO, USA) was prepared with distilled water, remaining under agitation until the complete dissolution of the polymer. Then, the solution containing the nitrates was added slowly to the PVP solution, under stirring for 2 h at 300 K. Hybrid fibers composed of metal–nitrate precursors and PVP (as-spun fibers) were produced using the previously prepared solution and a home-made solution blow spinning (SBS) device (Figure 1). A working distance of 50 cm, an injection rate of 4.5 mL/h, and an air pressure of 0.21 MPa were used in the spinning process. Solvent evaporation was improved by placing a tubular oven between the SBS nozzle and the fiber collector. This heating zone was set to 80 °C. The as-spun fibers were annealed in two stages in an oven using an alumina crucible to produce ferrite materials. The heating schedule employed a first stage of calcination at 150 °C, followed by a second stage at 500, 600, or 700 °C. In both stages, the dwell time was 2 h with a heating rate of 5 °C/min. The nomenclatures used to identify the nanofibers after calcination at 500, 600, and 700 were Ni500, Ni600, and Ni700, respectively.

### 2.2. Sample Characterizations

A Scanning Electron Microscope (Shimadzu, SSX-550, Kyoto, Japan and TESCAN, VEGA 3, Brno, Czech Republic) operating with a 20 kV voltage was used to characterize the morphology of the Ni500, Ni600, and Ni700 fibers. Image J (National Institutes of Health, USA, version 1.51k 1.6.0_24) was used to statistically analyze the average diameter of more than 100 fibers of each sample. The crystalline structure of the fibers after calcination was investigated by X-ray diffraction (Shimadzu, XRD-6000, Kyoto, Japan) with a Cu-K_alpha_ radiation source (λ = 1.5404 Å), scan 2theta (25–80°) and an angle step of 0.02°. Rietveld refinement was performed using the MAUD (Materials Analysis Using Diffraction, versio 2.999) software. The magnetic properties of the produced materials were recorded at room temperature using a commercial vibrating sample magnetometer (VSM) (Lake Shore, model 7400, San Diego, CA, USA), with a magnetic field in the ±15 kOe range and a step field of 80 Oe. N_2_ adsorption/desorption isotherm analysis (Quantachrome ASiQwin, version 3.01) was used to determine the specific surface area by the Brunauer–Emmett–Teller (BET) model and to determine the average pore size by the Barrett–Joyner–Halenda (BJH) model.

### 2.3. Adsorption Experiments

The Congo red (CR) dye adsorption experiments onto NiFe_2_O_4_ were conducted by adding 1.0 g/L of the adsorbent to 10 mL of CR solution with a concentration of 20 mg/L in a glass flask. The suspensions were agitated in the dark at a temperature of 300 K and at 180 rpm using a Shaker incubator (Nova Técnica, NT 735, Piracicaba, Brazil). Then, an aliquot of the suspension was withdrawn from the glass flask periodically. The fibers were separated from the suspension by centrifugation. The CR concentration of the residual solution was measured by a UV-vis spectrophotometer (Shimadzu, UV-1800, Kyoto, Japan) at a wavelength of 501 nm and by scanning in the 400–800 nm range. The adsorption capacity of the fibers at time *t* (*Q_t_*) and the efficiency removal (*R*) percentage were determined by Equations (1) and (2), respectively. Additionally, the Ni500 (1.0 g/L) adsorptive performance was investigated using different CR concentrations (5, 10, 20, 50, 100 mg/L) and doses (0.5, 1.0, 2.0 g/L).

(1) $$Q_{t}=\frac{\left(C_{o}-C_{t}\right)}{m}*V$$

(2) $$R=\left(C_{o}-C_{e}\right)*\frac{100\%}{C_{o}}$$

where *C_o_* (mg/L), *C_t_* (mg/L), and *C_e_* (mg/L) are the initial concentration, concentration at time *t* and at equilibrium, respectively, *m* (g) is the adsorbent mass, and *V* (L) is the volume of the CR solution.

Kinetic analysis was performed using the linear intraparticle diffusion model (Equation (3)) and the nonlinear Langmuir (Equation (4)) and Freundlich (Equation (5)) models for different concentrations and sorbent doses.

(3) $$Q_{t}=K_{id}t^{\frac{1}{2}}+C$$

(4) $$\log\left(Q_{e}-Q_{t}\right)=logQ_{e}-\frac{K_{1}}{2.303}t$$

(5) $$\frac{t}{Q_{t}}=\frac{1}{K_{2}Q_{e}^{2}}+\frac{t}{Q_{e}}t$$

where *Q_t_* and *Q_e_* (mg/g) represent the capacity of sorption in time *t* (min) at a quasi-equilibrium state (about 240 min), $K_{id}$ (mg/g min^1/2^) represents the internal diffusion coefficient, *C* (mg/g) is the boundary layer thickness, and *K*_1_ (min^−1^) and *K*_2_ (g.mg^−1^ min^−1^) are the adsorption rate constants of the pseudo-first-order and the pseudo-second-order kinetic models, respectively.

The sorbed material–sorbent relationship was studied using sorption isotherm models. The isotherm models used to analyze the equilibrium data were Langmuir (Equation (6)), Freundlich (Equation (7)), Brouers–Sotolongo (Equation (8)), Sips (Equation (9)), and Temkin (Equation (10)) models:

(6) $$Q_{e}=Q_{m}\frac{K_{L}C_{e}}{1+K_{L}C_{e}}$$

(7) Qe=KFCe1n

(8) $$Q_{e}=Q_{m}\left(1-e\left(-K_{W}C_{e}^{\alpha }\right)\right)$$

(9) $$Q_{e}=\frac{Q_{m}{\left(K_{S}C_{e}\right)}^{n}}{1+{\left(K_{S}C_{e}\right)}^{n}}$$

(10) $$Q_{e}=\frac{RT}{b}\ln\left(K_{T}C_{e}\right)$$

where *Q_m_* and *Q_e_* (mg/g) are the maximum and equilibrium capacity of sorption, respectively, *C_e_* is the equilibrium CR concentration, *K_L_* (L/mg) is the Langmuir constant, *K_F_* (mg/g) (L/mg)^1/n^ is the Freundlich constant and *n*^−1^ is the heterogeneity surface, which indicates the relative distribution of energy and the heterogeneity of the adsorbate sites. *K_W_* represents the Brouers–Sotolongo constant, α is a constant that measures the surface energy heterogeneity, *K_S_* (L/mg) is the Sips constant related to sorption affinity, *R* is the universal gas constant (8.314 J/mol K), *T* is the absolute temperature (298.15 K), and *K_T_* (L/g) and *b* represent the Temkin constants.

## 3. Results and Discussion

### 3.1. Microstructure Characterization

**Figure 2:**
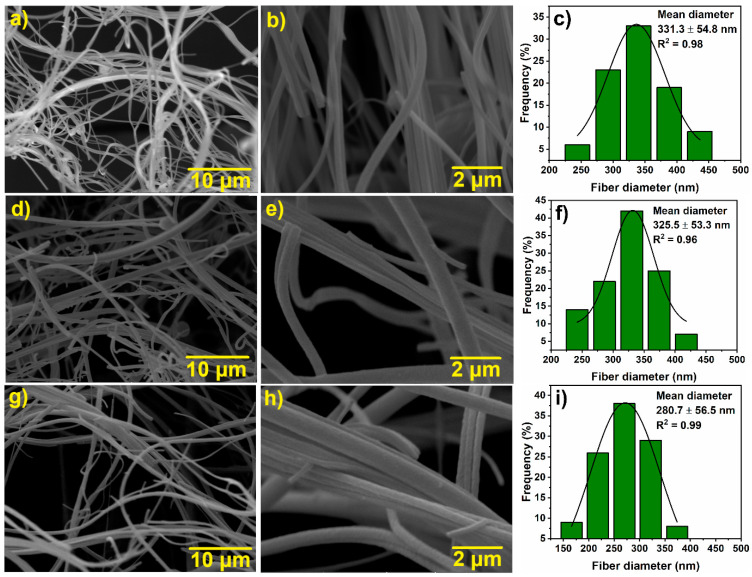
SEM micrographs of the Ni-ferrite fibers: Ni500 (**a**,**b**), Ni600 (**d**,**e**), and Ni700 (**g**,**h**). Gaussian fit diameter distribution histograms of the Ni500 (**c**), Ni600 (**f**), and Ni700 (**i**) fibers.

Figure 2 shows SEM micrographs of the Ni500 (Figure 2a,b), Ni600 (Figure 2d,e), and Ni700 (Figure 2g,h) nanofibers with 5000× and 20,000× magnifications. The micrographs show continuous fibers with several micrometers in length, a rounded transverse cross-section, and a rough surface. The diameter distribution histograms fitted to a Gaussian model are presented in Figure 2c,f,i. These histograms show that the average fiber diameters are similar, showing little variation from about 331 to 281 nm with the increase in the temperature of calcination. There is, however, an increasing predominance of fibers with smaller diameters as the calcination temperature rises from 500 °C to 700 °C, as evidenced in the histograms.

**Figure 3:**
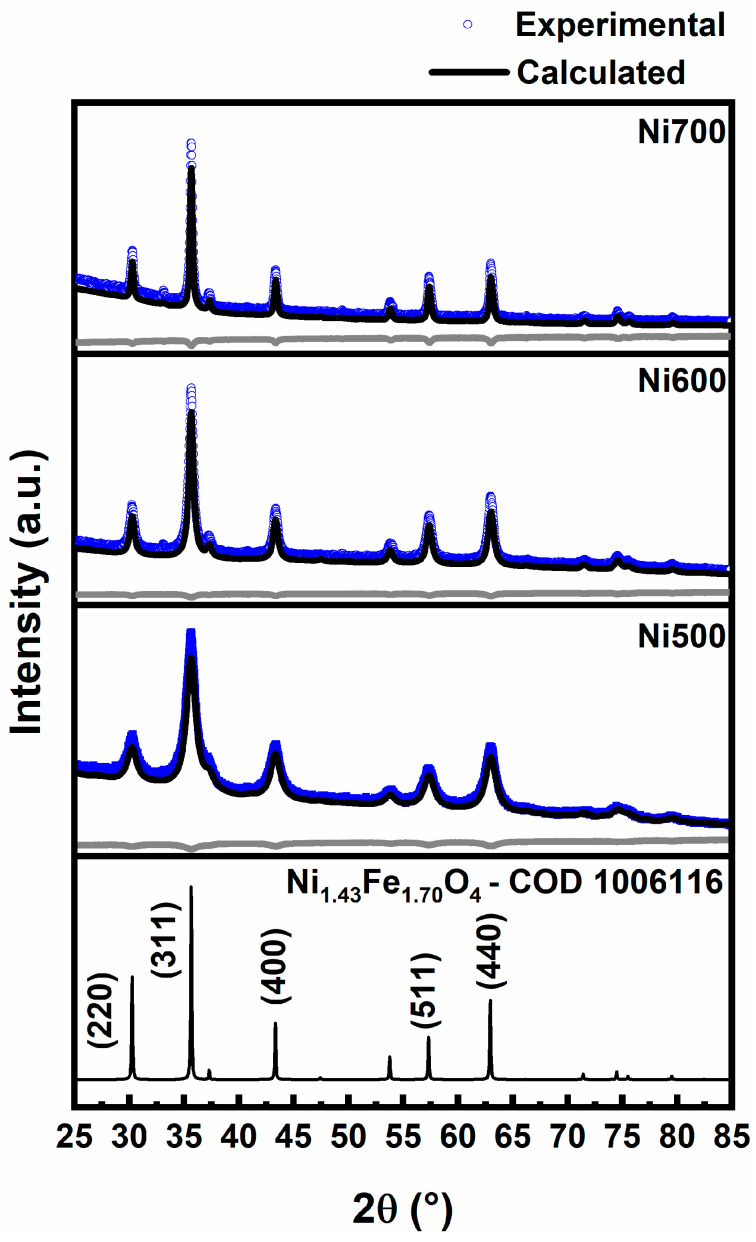
X-ray diffraction patterns of the Ni500, Ni600, and Ni700 fibers.

Figure 3 shows the XRD diffraction patterns of the Ni500, Ni600, and Ni700 fibers, revealing characteristic peaks of the cubic–spinel structure belonging to the *Fd* $\bar{3}$ *m* space group with phase Ni_1.3_Fe_1.7_O_4_ (COD 1006116). The main diffraction peaks centered at about 30.3°, 35.7°, 43.4°, 57.4°, and 63.0° correspond to the reflection planes (220), (311), (400), (511), and (440), respectively. The XRD patterns do not show any peaks related to additional phases. As the temperature increases from 500 °C to 700 °C, the XRD peaks become narrower, reflecting the relief of internal strain within the crystal structure [29] and the increase in crystal size and perfection [30]. Rietveld refinement analysis of the XRD was performed to estimate the structural parameters of the annealed fibers. As expected, the crystallite size (*D*) of the Ni500 (10.84 nm), Ni600 (24.10 nm), and Ni700 (56.98 nm) fibers increased with the increase in the calcination temperature. Yet, the lattice parameter (*a*) remained unchanged at 8.34 Å for all the nanofiber samples studied. Additionally, heating the Ni_1.3_Fe_1.7_O_4_ fibers at 500, 600, and 700 °C did not cause high stress in the crystal lattice (about 10^−4^). The resulting goodness of fit values, *R*^2^ < 2, shows a high level of agreement between the data and the revised models.

**Figure 4:**
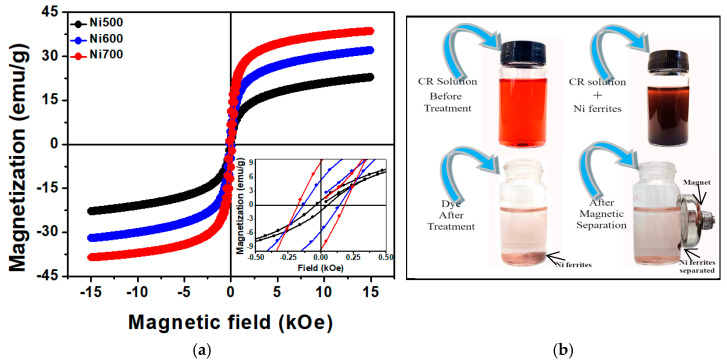
(**a**) Magnetic hysteresis loops for the Ni500, Ni600, and Ni700 samples at room temperature. (**b**) Efficient removal of CR dye and withdrawal of fibers using a magnet.

Figure 4a shows the room-temperature magnetic hysteresis loops for the samples annealed at 500, 600, and 700 °C. The symmetrical loops and low magnetic coercivity of the hysteresis loops, characteristic of nickel ferrites, are present in all situations. The saturation magnetization (*M_s_*) was obtained using the law of approach to saturation [31] and the coercivity field (*H_C_*) and remanence magnetization (*M_r_*) were obtained from the experimental curve (see the inset of Figure 4a). Table 1 summarizes the resulting outcomes.

Specifically, the saturation magnetization rises with the calcination temperature, which might explain the higher magnetic volumes associated with the larger particle sizes [32]. At 300 K, the saturation magnetization of samples of Ni_1.3_Fe_1.7_O_4_ annealed to 500, 600, and 700 °C is reasonable and comparable with that of NiFe_2_O_4_ materials [33,34]. However, the difference between experimental and theoretical values of *M_s_* (50 emu/g) can be attributed to size effects [35] or to the mass contribution of amorphous non-magnetic carbon present around particles and inside the nanofibers.

The coercivity field increases from 46 to 179 Oe as the calcination temperature increases, in agreement with reports in the literature [36,37,38]. The higher effective anisotropy energy, which is dependent on the size of the nanoparticles, could have caused an increase in coercivity. The reduced remanence *M_r_/M_S_* values are far below 0.5 and depend strongly on grain size, as previously reported for NiFe_2_O_4_ nanoparticles [33,39,40]. Therefore, Ni_1.3_Fe_1.7_O_4_ fibers annealed at 500, 600, and 700 °C show a favorable response to an external magnetic field. Thus, magnets can separate fibers from dispersion after the pollutant adsorption process, facilitating the potential application and reuse of the adsorbent (see Figure 4b). Because Ni700 has a higher *M_s_* value than Ni500 and Ni600 do, it has a better magnetic response to an external magnetic field, resulting in faster Ni700 attraction and separation from the treated solution.

**Figure 5:**
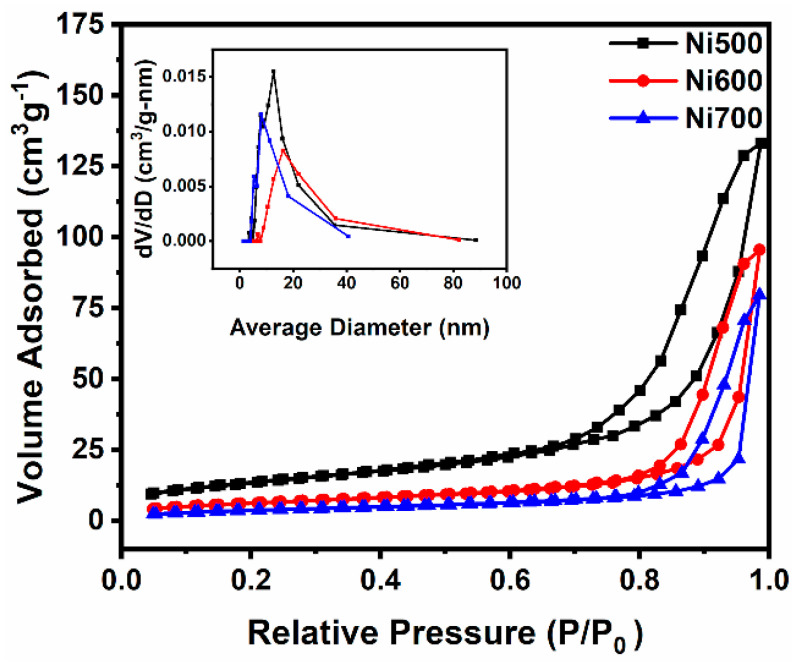
Adsorption/desorption isotherms of the Ni500, Ni600, and Ni700 fibers. The inset shows the pore size distribution (dV/dD) curves.

Figure 5 shows the N_2_ adsorption/desorption isotherms of the Ni500, Ni600, and Ni700 fibers. According to the IUPAC classification, all the annealed fibers presented type IV isotherms with H3 hysteresis loops. The type IV isotherm is related to capillary condensation in mesopores with monolayer–multilayer adsorption [41,42], and the H3 hysteresis loop characterizes the absence of limiting adsorption at high relative pressures (P/P_0_). The parameters obtained from adsorption/desorption isotherms and corresponding average pore sizes are summarized in Table 2.

In the analyzed temperature range, the surface area gradually decreases with crystallite size from 48.62 to 13.87 m^2^/g. However, the specific surface area values obtained here are comparable to those reported for nanostructured ferrites, such as CaFe_2_O_4_ [43], ZnFe_2_O_4_ [44], CoFe_2_O_4_ [45], and NiFe_2_O_4_ [46]. Additionally, pore volume and the specific surface area decrease with the calcination temperature, although the average pore diameter presents two behaviors. First, it increases from 127.00 Å (range: 27–847 Å) for the Ni500 fibers to 160.99 Å (range: 27–783 Å) for the Ni600 sample and then decreases to 80.50 Å (range: 27–773 Å) for the Ni700 sample (see inset of Figure 5). The average pore diameter behavior could be attributed to the mixture of smaller and larger pore sizes present along the fibers. In general, as the calcination temperature increases, the fiber becomes denser, given the sintering process, decreasing the surface area and pore volume, as shown in Table 2. The smaller pores likely vanish around 600 °C, leaving only the larger pores and, consequently, resulting in a higher average pore diameter. The large pores decrease in size at higher calcination temperatures, which cause sintering and densification.

### 3.2. Adsorption Experiment

#### 3.2.1. Effect of Contact Time and Initial Concentration

**Figure 6:**
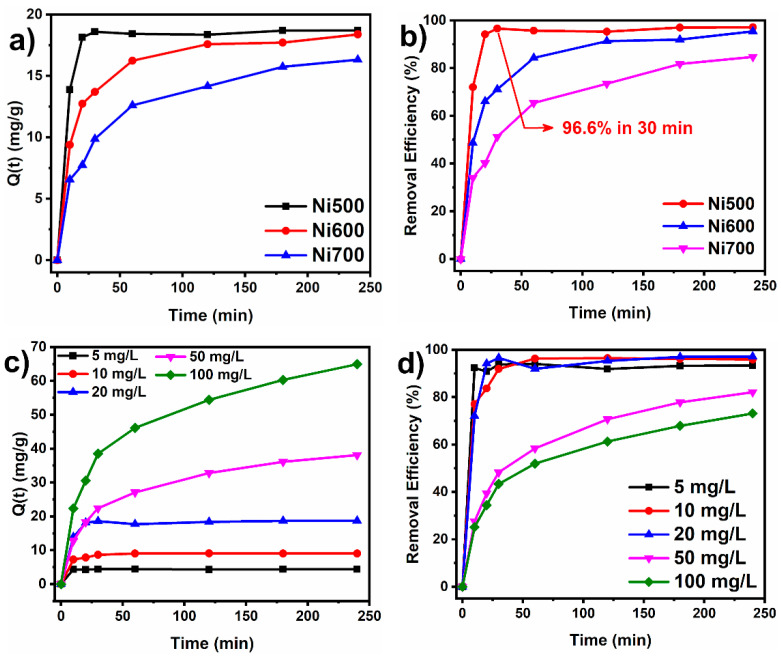
(**a**) Effect of contact time on the adsorption capacity, (**b**) removal efficiency of the Ni500, Ni600, and Ni700 sorbents, (**c**) effect of initial dye concentration on the adsorption capacity for the Ni500 sorbent, and (**d**) removal efficiency of the Ni500 sorbent at different initial dye concentrations.

The effect of the contact time and the initial dye concentration on the adsorption capacity of the nickel ferrite fibers was investigated to understand the equilibrium conditions between the dye solution and the sorbent. Figure 6a,b show the adsorption capacity and the removal efficiency (%) of CR (20 mg/L concentration) using a dose of 1 g/L of Ni500, Ni600, and Ni700 in 10 mL of CR solution. The sorbent dose of 1 g/L was chosen for the experiments because it provided the best relation between sorption capacity and cost-effectiveness. It can be observed that the degree of sorption increases as the contact time increases. This is caused by the initial abundance of vacant sorption sites in the early stages of the process that allow for faster adsorption. After 120 min, the degree of sorption almost stabilizes, and the sorption capacity reaches a quasi-equilibrium value, reflecting the equilibrium between the amount of dye effectively sorbed and that present in the solution. The nearly constant sorption capacity indicates a saturation of active sites in the nickel ferrite fibers. At the saturation level, the remaining vacant sites may be difficult to reach due to the presence of repulsive forces between the CR molecules in the solution and the molecules adsorbed on the surface [47]. Notably, the adsorption capacity and the CR removal efficiency of the Ni500 fibers are higher than those of the Ni600 and Ni700 fibers. The adsorption capacity of the Ni500, Ni600, and Ni700 fibers at *t* = 30 min were 18.6, 13.7, and 9.8 mg/g, respectively. Also, the Ni500 fibers presented 96.6% efficiency in the removal of CR in only 30 min compared to the efficiencies of 71.1% and 51.3% achieved by the Ni600 and Ni700 sorbents. Moreover, as suggested in Figure 5, the Ni500 fibers reach sorption equilibrium after only 30 min of the experiment. The adsorption capacity and removal efficiency decreased for the Ni700 sorbent following the decrease in surface area and average pore size.

Figure 6c,d show the effect of the initial concentration on the adsorption capacity and removal efficiency. As can be inferred from Figure 6c, the adsorption capacity at t = 240 min increases from about 4.4 mg/g to 65 mg/g as the initial CR dye concentration rises from 5 mg/L to 100 mg/L. At low concentrations, the number of active sites available is far superior to the quantity of sorbate in the solution. Thus, the sorption process is considered independent of the initial dye concentration [48]. The higher sorption at higher concentrations reflects an improved driving force and faster mass transfer [49]. It has also been observed that at concentrations above 20 mg/L, the adsorption capacity decreases compared to the dye concentration, likely due to saturation of the adsorbent surface [50].

#### 3.2.2. Effect of Adsorbent Mass

**Figure 7:**
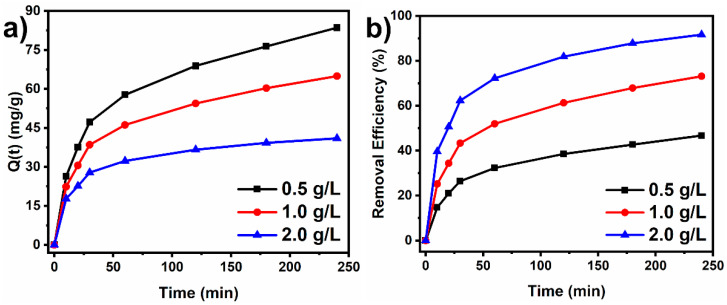
Effect of adsorbent mass on the adsorption capacity (**a**) and removal efficiency (**b**) of the Ni500 fiber.

The adsorbent dosage is a key factor that influences the adsorption process of a dye. The adsorption capacity and removal efficiency of the Ni500 sorbent were evaluated by varying the sorbent dosage from 0.5 g/L to 2 g/L. The experiments were conducted using a concentration of the CR solution of 100 mg/L and stirring rate of 180 rpm, at pH 7 and at room temperature (25 °C). Figure 7a,b present the adsorption capacity and the removal efficiency for three adsorbent doses (0.5 g/L, 1.0 g/L, and 2.0 g/L). Increasing adsorbent dose led to an increase in the CR removal efficiency from about 46.7% to 91.6% (Figure 7b). This was caused by the larger number of adsorption sites available for dye adsorption as the sorbent dose increased in the solution [22]. Contrarily, the adsorption capacity suffered a drastic decrease with increases in sorbent dose. Increasing the sorbent dose causes an increase in the specific active surface area of the sorbent available, and only a limited number of CR molecules in the solution can adsorb at those sites. Therefore, at higher sorbent doses, some active sites remain free, leading to a decrease in the adsorption capacity from 83.5 mg/g to 40.9 mg/g.

#### 3.2.3. Kinetic Study

**Figure 8:**
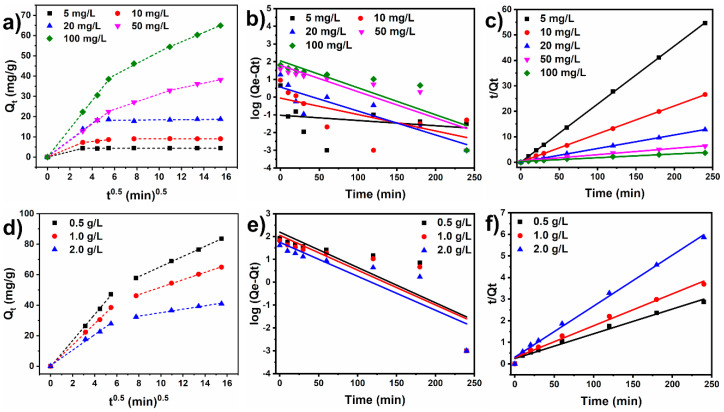
Plots of *Q*_t_ vs. *t*^1/2^ for the intraparticle diffusion model for initial dye concentrations from 5.0 mg/L to 50.0 mg/L (**a**) and for different sorbent doses (**d**). Plots of *Q_t_* vs. *t* for the pseudo-first-order (**b**,**e**) and pseudo-second-order (**c**,**f**) models for initial dye concentrations from 5.0 mg/L to 50.0 mg/L and sorbent doses from 0.5 g/L to 2.0 g/L.

The mechanisms governing the CR dye adsorption kinetics on the Ni500 fiber were investigated by fitting the experimental data with the linear intra-particle diffusion model (Equation (3)) and nonlinear models: the pseudo-first-order (Equation (4)) and pseudo-second-order (Equation (5)) models. Figure 8 exhibits the data plots of the kinetic models, and Table 3 and Table 4 summarize the kinetic parameters.

According to Table 3 and Table 4, the pseudo-first-order model did not fit well with the experimental data, as evidenced by the low linear regression coefficients (*R*^2^). The resulting goodness of fit values demonstrates a high degree of accordance between the kinetic pseudo-second-order model and the experimental data, with *R*^2^ > 0.983. As can be observed, the experimental adsorption capacity values approximate those obtained from the pseudo-second-order model. The pseudo-second-order model shows that the chemisorption of CR dye molecules onto adsorbent active sites may be the rate-controlling step at which CR adsorbs onto the fiber [51]. In the chemisorption of organic molecules, valence forces are involved through the exchange or sharing of electrons [13]. Furthermore, the rapid initial adsorption may have been caused by physical interactions with the adsorbent surface. These findings contribute to a better understanding of the interaction between the molecules of Congo red dye and the surface of the NiFe_2_O_4_ fibers, which could be of practical use in optimizing water remediation systems based on adsorption phenomena.

The data were fitted to the linear intraparticle diffusion model to study the diffusion mechanisms involved in the sorption process and the rate-limiting step. Figure 8a shows that, at low initial concentrations (5 to 20 mg/L), the kinetic data tends to exhibit two linear segments, while three segments could be found above 50 mg/L. The low dye concentration is rapidly sorbed by the nickel ferrite sorbent in the first step because the amount of CR molecules available for adsorption is scarcer. In this case, the first stage represents rapid dye diffusion through the water to the sorbent surface, while the second stage corresponds to the sorption equilibrium. On the other hand, for higher concentrations, namely 50 mg/L and 100 mg/L, there is an intermediate linear segment ascribed to the diffusion of CR through the sorbent pores, which is slower intraparticle diffusion. Increasing the sorbent dose did not seem to affect the intraparticle kinetic behavior, as evidenced by the only two linear segments in Figure 8d. The *K_id_*_1_ values were superior to the *K_id_*_2_ values, which indicates that intraparticle diffusion was the rate-controlling step of the sorption process. The *K_id_*_1_ and *K_id_*_2_ values increased as the initial concentration went from 5 mg/L to 100 mg/L owing to the intensification of the driving forces in the medium.

#### 3.2.4. Adsorption Isotherm Studies

**Figure 9:**
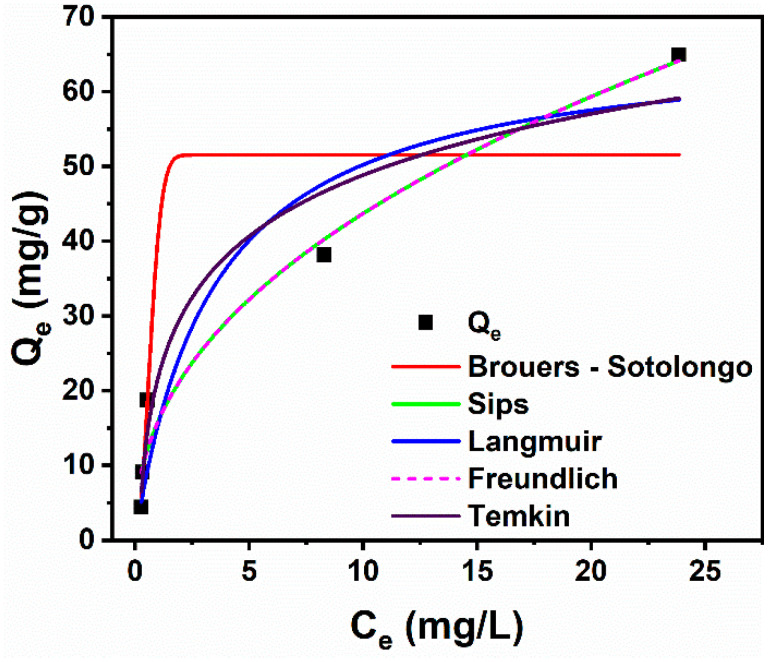
Plots of *Q_e_* vs. *C_e_* and fittings with the Brouers–Sotolongo, Sips, Langmuir, Freundlich, and Temkin isothermal models.

Isotherm models provide a deeper understanding of the interaction mechanisms between sorbents and sorbates, the affinity of sorption sites, and sorbent surface properties [13,52]. The adsorption behavior of CR dye on the Ni500 fibers was investigated by the nonlinear fitting of the experimental data with the Langmuir, Freundlich, Brouers–Sotolongo, Sips, and Temkin models. The corresponding fitted curves showing the dependence of *Q_e_* on *C_e_* are shown in Figure 9. Table 5 presents the theoretical isothermal parameters obtained for each model. The regression coefficients (*R*^2^) indicate the models that best fit the experimental data. According to Table 5, the Freundlich (*R*^2^ = 0.97), Sips (*R*^2^ = 0.97), Temkin (*R*^2^ = 0.95), and Langmuir (*R*^2^ = 0.93) models present the highest R^2^ values, suggesting that these models fit the experimental data more adequately and, therefore, better describe the sorption of CR dye onto NiFe_2_O_4_ fibers. The data follows the Freundlich isotherm well, indicating that the adsorption sites have a heterogeneous character. The Freundlich exponent *n*, whose inverse 1/*n* = 0.4425 is intermediate between 0 and 1, also implies that the nature of the sorbent surface is heterogenous to some degree. The data that fit the Sips model well indicate that both Freundlich and Langmuir model adequately describe the sorption process. The system exhibits the complex nature of both monolayer adsorption at high concentrations and heterogeneous adsorption at low concentrations [53]. Furthermore, the Sips exponential factor *n* is greater than 1, which suggests that the sorption did not follow only the Freundlich isotherm model. This is also confirmed by the high regression coefficient encountered for the Langmuir model (*R*^2^ = 0.93). The Langmuir isothermal model best approximated the experimental adsorption capacity, indicating that the data followed the Langmuir model to some extent. The value of the Temkin isotherm constant *b*, which represents the heat of adsorption, was found to be 209.52 kJ.mol^−1^, indicating that the adsorption process was exothermic [54]. Interestingly, the Ni500 fiber adsorption capacity for CR dye is found to be even higher than others reported, for instance NiO nanosheets (36.1 mg/g) [55], hollow Sn-Fe_2_O_4_ nanospheres (16.6 mg/g) [56], ZnO/activated carbon (51 mg/g) [57], and ZnO nanoparticles (9.6 mg/g) [58].

Adsorption capacity is determined by the sorbent material, possible surface modifications, initial dye concentration, and the adsorbent’s functional groups. Ni ferrite is well known for its enhanced adsorption properties towards organic additives [59,60], as well as due to its large surface area and the presence of functional groups (HOH, Fe-OH) on its surface [61]. The surface of Ni_1,43_Fe_1,7_O_4_ also has positive charges related to metallic cations (Ni^2+^ and Fe^3+^) that can bind to the two anionic sulfonate groups of the Congo red molecule. The electrostatic attraction between these elements contributes to an improved heat of adsorption. Furthermore, the large pores and specific surface area of BET fibers provide a greater number of active sites for additive adsorption, contributing to better CR removal from suspensions.

## 4. Conclusions

Nickel ferrite nanofibers were successfully produced using the solution blow spinning method. The nanofibers annealed at 500 °C demonstrated 96.6% removal of Congo red from water after only 30 min of the experiment and a maximum sorption capacity of 65 mg/g (dose of 1.0 g/L and 100 mg/L initial concentration), enhancing adsorption performance compared with that of Ni-ferrite annealed at 600 and 700 °C. The cubic–spinel structure of nickel ferrite, its porous nature, and the nanostructure of its fibers were confirmed by XRD, BET, and SEM analyses. The saturation magnetization (*M_s_*) confirmed the magnetic nature of the fibers, which favorably respond to an external magnetic field, facilitating the separation from a treated solution. The presence of pores in the fibers, as well as the electrostatic attraction between cations on the surface of the adsorbent (Ni^2+^ and Fe^3+)^ and anionic sulfonate groups of the dye, contributed to the adsorption mechanism. The experimental adsorption data were better fitted to the pseudo-second-order kinetic model and the Langmuir, Freundlich, Sips, and Temkin isotherm models. This suggests that a highly complex adsorptive nature, including chemisorption, monolayer adsorption at high concentrations, and heterogeneous adsorption at low concentrations, carried out the adsorption process. Finally, despite the high efficiency of Congo red removal shown here, further studies should be conducted to enhance adsorption capacity, reusability, and magnetization strength for easier separation. Additionally, exploring hybrid materials or functionalized surfaces could further increase the material’s affinity for Congo red dye, ensuring more effective removal at lower concentrations.

## Figures and Tables

**Table 1:** Magnetic parameters of the Ni-ferrite fibers.

Sample	M_s_ (emu/g)	M_r_/M_s_	H_c_ (Oe)
Ni500	24.84	0.04	46
Ni600	36.07	0.15	141
Ni700	41.68	0.22	179

**Table 2:** N_2_ adsorption/desorption parameters.

Sample	Surface Area (m^2^/g)	Pore Volume (cm^3^/g)	Average Pore Diameter (Å)
Ni500	48.62	0.21	127.00
Ni600	22.78	0.15	160.99
Ni700	13.87	0.13	80.50

**Table 3:** Kinetic parameters for the sorption of CR onto Ni500 fibers at various initial concentrations.

Parameters	Initial Concentration				
	5 (mg/L)	10 (mg/L)	20 (mg/L)	50 (mg/L)	100 (mg/L)
Qe, exp (mg/g)	4.42	9.07	18.71	38.11	64.95
Intraparticle diffusion model					
C_1_	3.140 × 10^−16^	6.28037 × 10^−16^	0.19450	−0.03843	0.01006
K_id1_	1.37243	2.29265	4.11773	4.09207	6.96631
R^2^	0.9999	0.9999	0.99611	0.99994	0.99931
C_2_	-	5.32316	-	12.17902	23.09579
K_id2_	-	0.59417	-	1.89278	2.88666
R^2^	-	0.98105	-	0.99826	0.99425
Pseudo-first order					
Qe,cal (mg/g)	0.095	0.883	3.698	62.011	112.466
K_1_ (min)	0.001	0.021	0.031	0.034	0.031
R^2^	0.092	0.31	0.755	0.733	0.688
Pseudo-second order					
Qe, cal (mg/g)	4.383	9.101	18.804	40.112	67.659
K_2_ (g/mg.min)	1.233	0.180	0.003	0.001	0.001
R^2^	0.999	0.999	0.999	0.987	0.986

**Table 4:** Kinetic parameters for the sorption of CR onto different sorbent doses.

Parameters	Initial Concentration		
	0.5 (g/L)	1.0 (g/L)	2.0 (g/L)
Qe, exp (mg/g)	83.56	64.95	40.97
Intraparticle diffusion model			
C_1_	−0.27225	0.0101	0.4410
K_id1_	8.55797	6.9663	5.0666
R^2^	0.99935	0.9993	0.9953
C_2_	32.2577	27.52446	23.87254
K_id2_	3.3088	2.43111	1.12636
R^2^	0.9994	0.99898	0.98979
Pseudo-first order			
Qe, cal (mg/g)	155.478	112.466	55.900
K_1_ (min)	0.035	0.035	0.034
R^2^	0.668	0.688	0.748
Pseudo-second order			
Qe, cal (mg/g)	87.260	67.659	42.224
K_2_ (g/mg.min)	0.005	0.008	0.002
R^2^	0.983	0.986	0.994

**Table 5:** Theoretical isotherms parameters of CR sorption onto the NiFe_2_O_4_ fibers.

Adsorption isotherm	Parameters	Values	R^2^
	Qe, exp (mg/g)	64.95	
Langmuir	Q_m_	67.29	0.936
	K_L_	0.294	
Freundlich	K_F_	15.82	0.972
	N	2.26	
Brouers–Sotolongo	Q_m_	51.53	0.852
	K_W_	1.512	
	A	2.006	
Sips	Q_m_	5.785	0.972
	K_S_	10.425	
	N	2.266	
Temkin	K_T_	6.202	0.948
	b	209.52	

## Data Availability

The original contributions presented in this study are included in the article. Further inquiries can be directed to the corresponding author.
