# Correction: Firmino et al. High-Efficiency Adsorption Removal of Congo Red Dye from Water Using Magnetic NiFe_2_O_4_ Nanofibers: An Efficient Adsorbent. *Materials* 2025, *18*, 754

**DOI:** 10.3390/ma18091891

**Published:** 2025-04-22

**Authors:** Hellen C. T. Firmino, Emanuel P. Nascimento, Keila C. Costa, Luis C. C. Arzuza, Rondinele N. Araujo, Bianca V. Sousa, Gelmires A. Neves, Marco A. Morales, Romualdo R. Menezes

**Affiliations:** 1 Laboratory of Materials Technology (LTM), Department of Materials Engineering, Federal University of Campina Grande, Campina Grande 58429-900, Brazil; emanueluepb@gmail.com (E.P.N.); keilaconceicaocosta@gmail.com (K.C.C.); luisarzuza179@gmail.com (L.C.C.A.); rondinelenunes93@gmail.com (R.N.A.); gelmires.neves@ufcg.edu.br (G.A.N.); 2 Graduate Program in Materials Science and Engineering, Federal University of Campina Grande, Campina Grande 58429-900, Brazil; 3 Department of Chemical Engineering, Federal University of Campina Grande, Campina Grande 58429-900, Brazil; biancavianaeq@gmail.com; 4 Department of Theoretical and Experimental Physics, Federal University of Rio Grande do Norte, Natal 59078-970, Brazil; marco.moralestorres@gmail.com

Keila C. Costa was not included as an author in the original publication [1]. The corrected Author Contributions statement appears here.

Author Contributions: Conceptualization, H.C.T.F., E.P.N., K.C.C., R.N.A., L.C.C.A. and R.R.M.; formal analysis and writing, H.C.T.F., E.P.N., K.C.C., R.N.A., L.C.C.A. and R.R.M.; review and supervision, R.R.M., B.V.S., M.A.M. and G.A.N.; project administration and funding acquisition, R.R.M. and G.A.N.; methodology, M.A.M. and R.R.M. All authors have read and agreed to the published version of the manuscript.

The authors state that the scientific conclusions are unaffected. This correction was approved by the Academic Editor. The original publication has also been updated.
